# Successful retrieval of a plastic bead from the airway of a child by flexible bronchoscopy and a balloon-tipped catheter: A case report and literature review

**DOI:** 10.1097/MD.0000000000012147

**Published:** 2018-09-14

**Authors:** Lina Wang, Li Zhang, Deli Li, Chunyan Li, Yan Wang, Man Gao, Hang Liang, Fanzheng Meng

**Affiliations:** Pediatric Department of Respiration II, The First Hospital of Jilin University, Changchun, China.

**Keywords:** balloon, flexible bronchoscopy, Fogarty catheter, foreign body, plastic bead, retrieval

## Abstract

**Rationale:**

Bronchial foreign body aspiration is a critical condition that jeopardizes the respiratory function of children. Prompt diagnosis and removal of the foreign body can reduce occurrence of foreign body complications and mortality. Aspiration of spherical plastic beads is rare, and the bead is difficult to retrieve.

**Patient concerns:**

An 8-year-old girl developed cough, transient throat wheezing, and intermittent cough after she accidentally inhaled a plastic bead 7 hours ago. Chest computed tomography scan revealed a round shadow 1.2 cm in diameter in the right main bronchus.

**Diagnoses:**

Foreign body in the right main bronchus.

**Interventions:**

Retrieval by balloon-tipped catheter via flexible bronchoscopy was undertaken.

**Outcomes:**

The bead was successfully retrieved and the child recovered uneventfully.

**Lessons:**

Foreign body aspiration in children constitutes a medical emergency in severe cases. Flexible bronchoscopy and balloon-tipped catheter retrieval can be used as an effective noninvasive treatment for aspiration of plastic beads.

## 1 Introduction

Airway foreign bodies pose a severe choking hazard for children, particularly those who are younger than 3 years of age. In the United States, it is estimated that 17,537 children aged 14 years or younger visited emergency departments for choking-related episodes in 2001, and many of these episodes were associated with candy/gum (19.0%) and coins (12.7%).^[1]^ A review of tracheobronchial foreign body aspiration in children in South Africa revealed that metal foreign bodies are the most common (44%) followed by plastic foreign bodies (21%).^[2]^ Furthermore, coins are the commonly aspirated foreign bodies (30%) followed by beads (8%). In China, on the contrary, nuts are the most common inhaled foreign bodies in children,^[3]^ with 1 report indicating that peanuts account for 87% of foreign bodies.^[4]^ Though a child with an airway foreign body may manifest paroxysmal cough, wheezing, and dyspnea upon initial presentation, he or she may also have atypical manifestations, rendering diagnosis difficult in the absence of a definite history of foreign body aspiration or ingestion and thus mandating a high index of clinical suspicion. In cases where a relatively large foreign body is inhaled such as round beads, apparent breathing difficulties, and asphyxiation may ensue, endangering the lives of the children and mandating emergency treatment. Removal of beads is challenging as beads are typically round or oval and slippery and in severe cases, extraction is undertaken of aspirated bead by open chest surgery under extracorporeal circulation.^[5]^ In the present study, we describe a case of plastic bead aspiration in a young girl that was successfully managed by bead retrieval via a balloon-tipped catheter and also provide a relevant literature review.

## 2 Case report

An 8-year-old girl was admitted to the Emergency Department of our hospital on April 10, 2015 because of accidental aspiration of a plastic bead 7 hours ago. A transient bout of coughing and labored breathing appeared, which spontaneously resolved without subsequent breathing difficulty. She had cough, transient throat wheezing, and intermittent cough. Examination at admission showed that the child was in a general good condition. No throat wheezing was present. There was no cyanosis and the inspiratory 3-concave sign was negative. The child had no breathing difficulty and hoarseness. Bilateral lung sounds were coarse on auscultation and chest expansion was largely symmetrical. No slap sound by a foreign body was heard. Chest computed tomography scan revealed a round shadow 1.2 cm in diameter in the right main bronchus, but was otherwise unremarkable. A diagnosis of foreign body in the right main bronchus was entertained.

**Figure 1:**
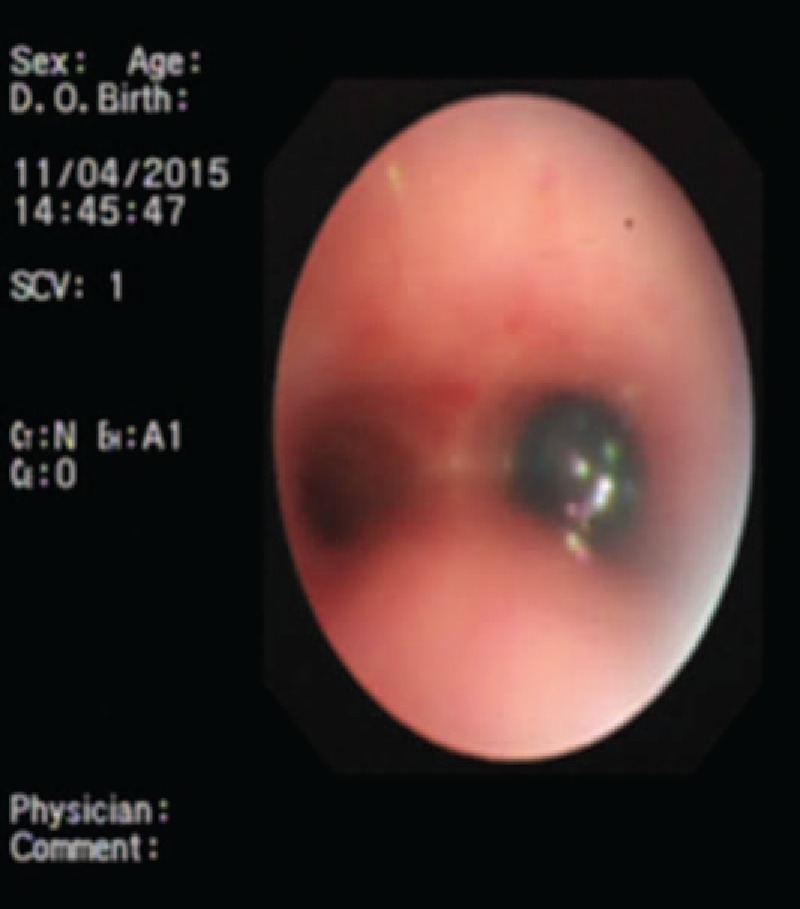
Flexible bronchoscopy reveals an impacted bead in the right bronchus.

**Figure 2:**
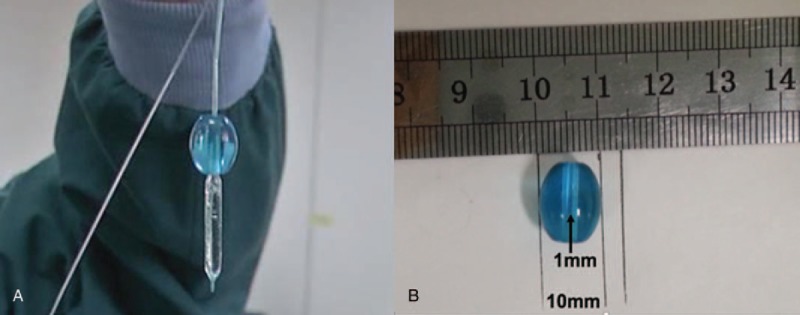
(A) A plastic bead is retrieved, and (B) the bead is 10 mm in size with a 1 mm central hole.

Emergency bronchoscopy was carried out upon admission, and the child underwent rigid bronchoscopy (STORZ) under general anesthesia for retrieval of the foreign body. A blue plastic bead was visualized directly under the rigid bronchoscope and was found to be closely impacted on the opening of the right main bronchus (Fig. 1). The bead was 1.0 cm in diameter with a central hole 1 mm in diameter and was not readily mobile and cannot be retrieved by forceps. A fiberoptic bronchoscope (Olympus BF-P260, Olympus medical corporation, Tokyo, Japan the external diameter 4.0 mm) was advanced through the laryngeal mask airway for re-exploration and a balloon-tipped catheter (lacrosse balloon catheter 4.0 mm × 20 mm) was entered at the same time via the maneuvering channel. The balloon was advanced through the central hole in the plastic bead and released and inflated after passing beyond the hole. The inflated balloon was 4 mm and after it was secured, it was pulled out along with the plastic bead (Fig. 2). The child had an uneventful recovery.

### 2.1 Literature review

We searched PubMed for published cases of bronchial aspiration of plastic beads using the keywords “bead,” “endobronchial foreign bodies,” “foreign body bronchus,” “Fogarty catheter,” and “balloon.” A total of 4 cases (including our current case) of bronchial aspiration of plastic beads published between January 1975 and November 2017 were identified in PubMed (Table 1). One report described the retrieval of the foreign body by surgery in a child in a critical condition.^[5]^ Their age ranged from 6 to 12 years. These children had varied manifestations. Chest X-ray findings were positive in most patients, including atelectasis, hyperventilation, and shadow of a foreign body. Except in the surgical case, all the plastic beads were removed using the “balloon bead technique.” Three cases underwent successful retrieval of the foreign body on the first attempt. In the remaining child, the balloon was improperly positioned and improper force was used to pull out the balloon, leading to rupture of the balloon. The plastic bead was retrieved on the second attempt under C-arm guidance. All the children recovered uneventfully after foreign body removal.^[5–11]^

**Table 1:**
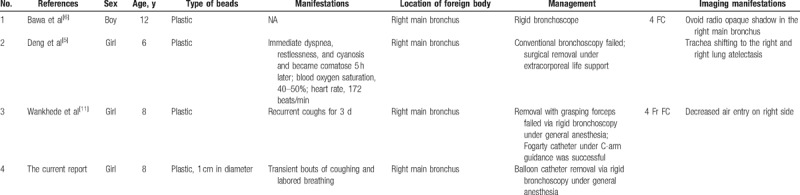
Literature review of pediatric cases with aspirated plastic beads.

## 3 Discussion

Foreign body aspiration in children constitutes a medical emergency in severe cases and is a major cause of accidental death in infants and preschool children.^[12]^ The earliest reported case involves the use of a balloon-tipped catheter to retrieve a carnival bead, but details are not provided.^[13]^ In 1959, Cantor described extraction of beads in 2 cases.^[14]^ Plastic beads may not be easily discerned radiologically that could delay diagnosis and prompt treatment.^[15]^ In the present study, we report a case of inhaled plastic bead that became impacted in the right main bronchus in an 8-year-old girl. The bead was successfully retrieved with the use of a balloon-tipped catheter and the girl recovered uneventfully.

Bronchial foreign body aspiration often occurs in children younger than 3 years of age and is more frequent in boys than girls.^[16,17]^ However, aspiration of beads is more common in girls, likely because girls use beads more often as toys. A foreign body is more commonly located in the right bronchus, which may be related to the anatomic features of the right main bronchus: the angle in the right bronchus is smaller while the diameter of the right bronchus is larger than that of the left bronchus. Bronchial foreign bodies mostly consist of peanuts, sunflower seeds, walnuts, and chicken bone in younger children, and pen caps and small toys or toy parts in older children.^[18,19]^ Plastic beads as foreign bodies are rare and difficult to retrieve, and no more than 10 cases have been reported over the previous decades.

Bronchial foreign body may cause partial or complete airway occlusion and lead to complications. The optimal time for retrieval of bronchial foreign body falls within 24 hours of foreign body aspiration and if retrieval is successful within this time frame, complications can be avoided.^[18]^ Pneumonia, bronchitis, and esophagobronchial fistula often ensue if the foreign body stays for more than 4 weeks.^[20]^ Therefore, prompt retrieval of the foreign body is critical. Williams et al^[21]^ categorize complications into mild and severe complications. The former includes decreased arterial blood saturation, bradycardia, bronchial spasm, and others. The latter includes larynx edema, pneumothorax, and sudden cardiac arrest. Ball-shaped foreign body-associated complications mainly depend on the diameter of the foreign body. A foreign body with a larger diameter may become fully impacted in the trachea and the main bronchi, and can directly cause death. If the foreign body becomes impacted on the main bronchus, in the absence of prompt retrieval, tissue compression and necrosis ensue, leading to atelectasis and lung collapse, eventually mandating surgery. The current case and the case reported by Landy et al^[10]^ experienced transient breathing difficulty upon foreign body aspiration. Deng et al^[5]^ found that the patient had immediate marked decline in blood oxygen saturation and metabolic acidosis and hypoxemia. Therefore, prevention of development of severe complications caused by ball-shaped foreign bodies requires prompt diagnosis and retrieval.

Foreign body clamp and tissue clamp are conventionally used for retrieving aspirated foreign body via bronchoscopy, and clasp clamp and visual foreign body clamp are used for removal of the foreign body via rigid bronchoscopy. Foreign body clamp and biopsy clamp can also be used for retrieving aspirated foreign body by flexible bronchoscopy; however, because of the slippery property, size and location of ball-shaped foreign body, the foreign body clamp is not very effective in retrieving a foreign body. Ullyot and Norman report the use of the Fogarty catheter to aid bronchoscopic removal of foreign bodies in 1968.^[22]^ It has also been reported that balloon-tipped catheter is effective in retrieving aspirated nuts, and blunt foreign bodies including peanuts, pumpkin seeds, olive nucleus, soybeans and pig bone,^[23]^ as well as downward facing thumbtacks.^[24]^

Balloon-tipped catheter is also considered a good option for retrieving aspirated plastic beads. For plastic bead, because of its large volume and smooth surface, it may become fully impacted in the main airway and it is difficult to retrieve using clamp, freezing, or net. Because there is a hole in the center of the bead, a balloon-tipped catheter can be advanced over the hole and after the balloon is inflated, the bead can be pulled out. The literature on retrieval of plastic beads uses this method. Balloon-tipped catheter can be advanced via a rigid or flexible bronchoscope. The anterior segment of the flexible bronchoscope is more flexible and makes it easier to pass the catheter over the central hole in the bead and has better maneuverability than the rigid bronchoscope.

In addition, the size of the balloon-tipped catheter ranges from 2 to 4 mm and the balloon does not need to be inflated to the greatest diameter and it suffices as long as the balloon passes beyond the center hole of the bead. Meanwhile, the pressure of the balloon is smaller than that used for treatment and moderate force is applied to pull out the foreign body. The major complication of the procedure is balloon rupture, which is generally due to improper positioning of the balloon, undue pressure, repetitive pulling out, or use of excessive force for pulling out the foreign body. These can be avoided during maneuvering and, if necessary, the balloon can be released under guidance by C-arm.^[8,11]^

In conclusion, aspiration of a ball-shaped foreign body by children is a critical condition, and prompt foreign body retrieval and prevention of complications should be undertaken. Balloon-tipped catheter retrieval may offer a noninvasive effective treatment method.

## Author contributions

**Writing – original draft:** Lina Wang.

**Investigation:** Li Zhang, Deli Li, Yan Wang.

**Resources:** Chunyan Li, Man Gao, Hang Liang.

**Conceptualization:** Fanzheng Meng.

## References

[1] Centers for Disease C, Prevention Nonfatal choking-related episodes among children - United States, 2001. MMWR Morb Mortal Wkly Rep 2002;51:945–8. 12437033

[2] Sultan TA van As AB Review of tracheobronchial foreign body aspiration in the South African paediatric age group. J Thorac Dis 2016;8:3787–96. 28149578 10.21037/jtd.2016.12.90 PMC5227244

[3] Huankang Z Kuanlin X Xiaolin H Comparison between tracheal foreign body and bronchial foreign body: a review of 1,007 cases. Int J Pediatr Otorhinolaryngol 2012;76:1719–25. 22944360 10.1016/j.ijporl.2012.08.008

[4] Zhijun C Fugao Z Niankai Z Therapeutic experience from 1428 patients with pediatric tracheobronchial foreign body. J Pediatr Surg 2008;43:718–21. 18405721 10.1016/j.jpedsurg.2007.10.010

[5] Deng L Wang B Wang Y Treatment of bronchial foreign body aspiration with extracorporeal life support in a child: A case report and literature review. Int J Pediatr Otorhinolaryngol 2017;94:82–6. 28167019 10.1016/j.ijporl.2017.01.011

[6] Bawa M Kalawant A Vinod MS Unsuccessful retrieval of impacted foreign body bronchus: think about Fogarty catheter. Indian J Pediatr 2016;83:744–5. 26634268 10.1007/s12098-015-1951-8

[7] Berry BE Davis DJ Acree PW Aspiration of Mardi Gras beads: new method for removal by Fogarty catheter. J La State Med Soc 1975;127:257–8. 1165419

[8] Elsharkawy H Abd-Elsayed AA Karroum R Management challenges in the passing-through technique using a Fogarty catheter to remove an endobronchial foreign body from an infant. Ochsner J 2015;15:110–3. 25829892 PMC4365839

[9] Good GM Deutsch ES Method for removing endobronchial beads. Ann Otol Rhinol Laryngol 1998;107:291–2. 9557762 10.1177/000348949810700405

[10] Landy C Massoure PL Gauthier J Use of a Fogarty catheter after tracheobronchial inhalation of a bead. Trop Doct 2012;42:219–20. 23131750 10.1258/td.2012.120012

[11] Wankhede RG Maitra G Pal S Successful removal of foreign body bronchus using C-arm-guided Insertion of Fogarty catheter through plastic bead. Indian J Crit Care Med 2017;21:96–8. 28250606 10.4103/ijccm.IJCCM_148_16 PMC5330062

[12] Samarei R Survey of foreign body aspiration in airways and lungs. Glob J Health Sci 2014;6:130–5. 25363168 10.5539/gjhs.v6n7p130 PMC4796343

[13] Jesberg S A method of removing a bead from a bronchus. Laryngoscope 1926;36:917–8.

[14] Cantor JJ A method of removing a bead from a bronchus. An improvement in procedure. Laryngoscope 1959;69:1537–9. 13807497 10.1288/00005537-195912000-00007

[15] Saps M Rosen JM Ecanow J X-ray detection of ingested non-metallic foreign bodies. World J Clin Pediatr 2014;3:14–8. 25254180 10.5409/wjcp.v3.i2.14 PMC4173203

[16] Hamouda S Oueslati A Belhadj I Flexible bronchoscopy contribution in the approach of diagnosis and treatment of children's respiratory diseases: the experience of a unique pediatric unit in Tunisia. Afr Health Sci 2016;16:51–60. 27358613 10.4314/ahs.v16i1.7 PMC4915407

[17] Tang LF Xu YC Wang YS Airway foreign body removal by flexible bronchoscopy: experience with 1027 children during 2000-2008. World J Pediatr 2009;5:191–5. 19693462 10.1007/s12519-009-0036-z

[18] Haddadi S Marzban S Nemati S Tracheobronchial foreign-bodies in children: a 7 year retrospective study. Iran J Otorhinolaryngol 2015;27:377–85. 26568942 PMC4639691

[19] Saki N Nikakhlagh S Rahim F Foreign body aspirations in infancy: a 20-year experience. Int J Med Sci 2009;6:322–8. 19851473 10.7150/ijms.6.322 PMC2764343

[20] Saquib Mallick M Rauf Khan A Al-Bassam A Late presentation of tracheobronchial foreign body aspiration in children. J Trop Pediatr 2005;51:145–8. 15831667 10.1093/tropej/fmh103

[21] Williams A George C Atul PS An audit of morbidity and mortality associated with foreign body aspiration in children from a tertiary level hospital in Northern India. Afr J Paediatr Surg 2014;11:287–92. 25323175 10.4103/0189-6725.143129

[22] Ullyot DG Norman JC The Fogarty catheter: an aid to bronchoscopic removal of foreign bodies. Ann Thorac Surg 1968;6:185–6. 5748023 10.1016/s0003-4975(10)66012-2

[23] Chen X Chen Y Zhong C The efficacy and safety of airway foreign body removal by balloon catheter via flexible bronchoscope in children - a retrospective analysis. Int J Pediatr Otorhinolaryngol 2016;82:88–91. 26857322 10.1016/j.ijporl.2016.01.003

[24] Thornton CS Yunker WK Rigid bronchoscopy and balloon dilation for removal of aspirated thumbtacks: case series and literature review. Int J Pediatr Otorhinolaryngol 2015;79:1541–3. 26209351 10.1016/j.ijporl.2015.07.007

